# Trends in Emergency Department Visits for Contact Sports–Related Traumatic Brain Injuries Among Children — United States, 2001–2018

**DOI:** 10.15585/mmwr.mm6927a4

**Published:** 2020-07-10

**Authors:** Dana Waltzman, Lindsay S. Womack, Karen E. Thomas, Kelly Sarmiento

**Affiliations:** 1 Division of Injury Prevention, National Center for Injury Prevention and Control, CDC.

During 2010–2016, there were an average of 283,000 U.S. emergency department (ED) visits each year among children for sports and recreation–related traumatic brain injuries (SRR-TBIs); approximately 45% of these SRR-TBIs were associated with contact sports (*1*). Although most children with an SRR-TBI are asymptomatic within 4 weeks, there is growing concern about potential long-term effects on a child’s developing brain (*2*). This has led to calls to reduce the risk for traumatic brain injuries (TBIs) among child athletes, resulting in the introduction of state policies and the institution of safety rules (e.g., age and contact restrictions) for some sports programs. To assess changes in the incidence of ED-related SRR-TBI among children, CDC analyzed data from the National Electronic Injury Surveillance System–All Injury Program (NEISS-AIP) for the period 2001–2018. After more than a decade of increasing rates, the rate of contact sports–related TBI ED visits declined 32% from 2012 to 2018. This reduction was primarily the result of a decline in football-related SRR-TBI ED visits during 2013–2018. Decreased participation in tackle football (*3*) and implementation of contact limitations (*4*) were likely contributing factors to this decline. Public health professionals should continue to expand efforts to address SRR-TBIs in football, which is the sport with the highest incidence of TBI, and identify effective prevention strategies for all sports to reduce TBIs among children.

NEISS-AIP is operated by the U.S. Consumer Product Safety Commission and each year houses data on approximately 500,000 initial injury-related visits for patients treated in hospital EDs. Data are drawn from a nationally representative sample of hospitals that have been selected as a stratified probability sample (*1*). Data are weighted by the inverse probability of selection to provide national estimates.

SRR-TBIs included TBIs among children aged ≤17 years that occurred during organized and unorganized SRR activities. Children were classified as having a TBI if the primary body part injured was the head and the principal diagnosis was concussion or internal organ injury. Each case was initially classified into one of 39 mutually exclusive sports and recreation–related groups on the basis of an algorithm that considered both the consumer products involved (e.g., bicycles, swing sets, and in-line skating equipment) and the narrative description of the incident obtained from the medical record. SRR activities were collapsed into categories (i.e., contact sport, limited contact sport, noncontact sport, or recreation) based on previous studies (*5*). Cases were excluded if the injury was violence-related or if the person was dead on arrival or died in the ED.

Rates of SRR-TBIs per 100,000 population per year were calculated using U.S. Census Bureau population estimates as the denominator, stratified by sex and age group. Rates and 95% confidence intervals were calculated using SAS software (version 9.4; SAS Institute), accounting for sample weights and the complex survey design. Trends in SRR-TBI ED visit rates were evaluated using Joinpoint software (version 4.7.0.0, National Cancer Institute) (https://surveillance.cancer.gov/joinpoint/).

From 2001 to 2018 an estimated 3,888,020 SRR-TBI ED visits occurred in the United States for children aged <17 years. The rate of SRR-TBI ED visits per 100,000 population aged ≤17 years declined 27% from 2012 (411.1) to 2018 (298.8), primarily driven by a 32% decline in the rate of contact sports-related TBI ED visits from 189.9 in 2012 to 129.4 in 2018 (Figure 1). In addition, the rate of noncontact sports–related TBI ED visits declined from 98.9 in 2012 to 75.5 in 2018. Among contact sports, the highest rates of TBI ED visits in 2018 in children aged 5–17 years were for injuries sustained while playing football (72.4), basketball (46.6), and soccer (32.5) (Figure 2). The rate of football-related TBI ED visits in children aged 5–17 years declined 39% from 118.8 in 2013 to 72.4 in 2018, after increasing approximately 200% from 2001 (38.7) to 2013 (118.8). TBI-ED visits for basketball and soccer, the other two leading contact sports, did not decline significantly.

**FIGURE 1:**
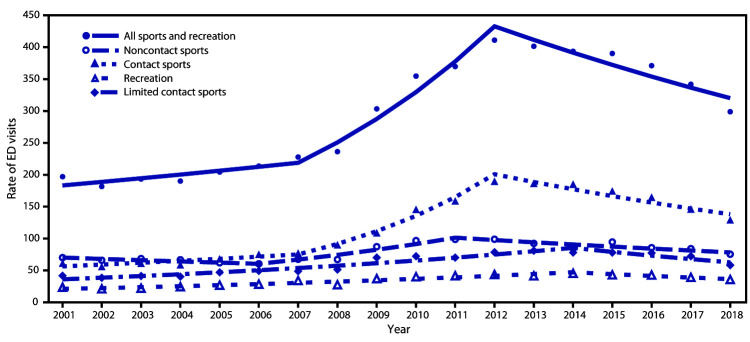
Trends* in rates^†^ of ED visits for nonfatal sports and recreation–related TBIs^§^ among persons aged ≤17 years, by type of activity^¶^ and contact level,**^,††,§§^ — National Electronic Injury Surveillance System–All Injury Program, United States, 2001–2018 **Abbreviations:** ED = emergency department; TBIs = traumatic brain injuries. * Symbols represent observed rates, and lines represent modeled rates. ^†^ Per 100,000 population. ^§^ All sports and recreation includes contact sports, limited contact sports, noncontact sports, and recreation. ^¶^ Recreation includes scooter riding, all-terrain vehicle riding, amusement attractions (rides and water slides [not swimming pool slides]), tobogganing/sledding, moped/dirt bike riding (includes other two-wheeled, powered, off-road vehicles and dune buggies), other recreation (includes nonpowder/BB guns, go-carts, personal watercraft, snowmobiling, camping, fishing, and billiards), miscellaneous recreation ball games (tetherball, kickball, and dodgeball), and other specified (gym/physical education class, archery, darts, curling, and mountain climbing). ** Contact sports include football, basketball, soccer, hockey (ice hockey, field hockey, roller hockey, and street hockey), combative sports (including boxing, wrestling, martial arts, and fencing), miscellaneous contact ball games (including lacrosse, rugby, and handball). ^††^ Limited contact sports include baseball, gymnastics (including cheerleading and dancing), skateboarding, softball, trampolining, horseback riding, volleyball, ice skating, inline/roller skating, and other limited contact sports (including snow skiing, snowboarding, water skiing, and surfing). ^§§^ Noncontact sports include playground, bicycling, swimming, exercise, golf (including injuries related to golf carts), track and field, racquet sports (tennis, badminton, and squash), and bowling.

**FIGURE 2:**
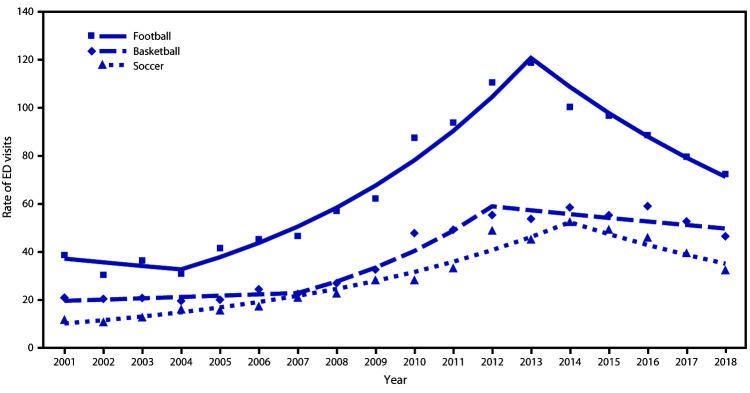
Trends* in rates**^†^** of ED visits for the three most common contact sports associated with nonfatal sports and recreation–related TBI among persons aged 5–17 years — National Electronic Injury Surveillance System–All Injury Program, United States, 2001–2018 **Abbreviations:** ED = emergency department; TBI = traumatic brain injury. * Symbols represent observed rates, and lines represent modeled rates. ^†^ Per 100,000 population.

The rate of contact sports-related TBI ED visits among children aged 10–14 and 15–17 years increased from 2001 to 2012 (Figure 3), then declined from 2012 to 2018. The pattern among children aged 5–9 years was similar: rates increased from 2001 to 2013 and then declined from 2013 to 2018. The estimated decline in annual percentage change from 2013 to 2018 differed by age group: declines of 8%, 5%, and 8% among children aged 5–9, 10–14, and 15–17 years, respectively.

**FIGURE 3:**
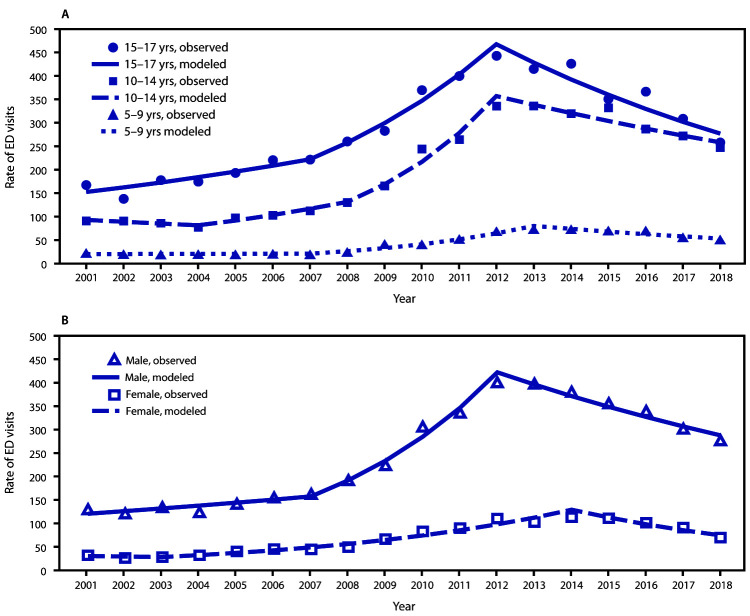
Trends* in rates**^†^** of ED visits for nonfatal sports and recreation–related TBI among persons aged 5–17 years, by age group (A) and sex (B) — National Electronic Injury Surveillance System–All Injury Program, United States, 2001–2018 **Abbreviations:** ED = emergency department; TBI = traumatic brain injury. * Symbols represent observed rates, and lines represent modeled rates. ^†^ Per 100,000 population.

A similar pattern of an initial increase in rate of contact sports–related TBI ED visits followed by a decline was observed by sex (Figure 3). From 2001 to 2012, the rate among males increased by approximately 200%, from 130.5 to 400.9 and among females, increased approximately 250% from 32.3 in 2001 to 113.5 in 2014. From 2012 to 2018, the rate among males declined 31%, to 277.3. From 2014 to 2018, the rate among females declined 38%, to 70.1.

## Discussion

This analysis found that from 2001 to 2018, approximately 3.8 million ED visits for SRR-TBIs occurred among children aged ≤17 years, with contact sports accounting for approximately 41% of these visits. After more than a decade of increasing rates, the rate of contact sports–related TBI ED visits declined 32% from 2012 to 2018. The increase in the early part of the study period might be associated with growing awareness and recognition of SRR-TBIs and therefore an increase in reporting (*6*); however, the reduction in the latter part of the study period was predominantly the result of a decline in ED visits related to football SRR-TBIs. These results highlight the importance of examining changes in sports-specific SRR-TBIs rates over time to understand the changing epidemiology of this injury.

Participation in organized youth football programs has declined 24% since 2010 (with a 12% reduction in participation from 2016 to 2017) (*3*), although it remains one of the most popular sports played by youths (*3*) and the sport with the highest rate of SRR-TBI. Approximately 25% of SRR-TBIs among children are attributed to football (*1*). Implementation of contact and tackling restrictions to reduce the risk for concussion and decreased participation in tackle football programs might also be contributing to the decline in football-related SRR-TBIs. Tackling is responsible for approximately two thirds of concussions and other TBIs among high school football players (*7*). Evidence suggests that contact restrictions and implementation of tackling techniques to reduce exposure to the head during a tackle (i.e., shoulder-style tackling) might reduce concussion risk by as much as 33% (*8*) and risk for overall head impact exposure by up to 42% (*9*). From 2012 to 2015, the National Federation of State High School Associations and its member states, as well as at least two large youth football programs, instituted guidelines to restrict the amount and frequency of full-contact drills during practices (*4*).

Most research on prevention of SRR-TBIs focuses on football and ice hockey and the effectiveness of sports safety equipment (e.g., helmets and mouthguards) (*2*). Studies on SRR-TBI prevention strategies for other contact sports (e.g., soccer and basketball) are limited. Although additional years of data might be needed to evaluate the trends in rates of SRR-TBI ED visits for nonfootball activities, the lack of evidence-based prevention strategies might be one reason for the absence of significant declines in the rates of SRR-TBI ED visits for nonfootball activities. Future research is also needed to identify effective prevention strategies for nonfootball activities to reduce SRR-TBIs among children.

The findings in this report are subject to at least six limitations. First, injury rates are underestimated because this study only included children treated in EDs. Many children with a TBI do not seek care in EDs (*10*) or do not seek care at all. Second, the estimates cannot be used to calculate relative risks for TBIs associated with SRR activities because there are limited data on national participation in SRR activities, especially for unorganized sports. Therefore, it is difficult to tell whether decreases in injuries result from interventions, decline in participation, or a combination of both. Third, because NEISS-AIP was not developed to identify specific diagnoses, actual TBIs might have been missed, and some injuries classified as TBIs might not have been. Fourth, because NEISS-AIP only included one diagnosis and body part injured, TBIs might be missed in cases where multiple injuries were present. NEISS-AIP did start including a second diagnosis in 2018; however, to be consistent with previous years, only the primary diagnosis was used for this study. Fifth, it cannot be determined whether the observed changes in the number of ED visits resulted from an actual change in incidence, care-seeking behaviors, or other reasons. Finally, although reported shifts in trends and corresponding annual percentage changes rely on analysis of aggregated survey data using Joinpoint software, a sensitivity analysis using case-level data in conjunction with complex survey software suggested qualitatively comparable findings.

Children participating in SRR activities are at risk for TBI. Therefore, expanding efforts to identify effective SRR-TBI prevention strategies will help ensure that children can continue to stay healthy and active.

**Summary**

**What is already known about this topic?**

During 2010–2016, an average of 283,000 U.S. emergency department (ED) visits per year for sports and recreation–related traumatic brain injuries (SRR-TBIs) occurred among children. Approximately 45% of these injuries were associated with contact sports.

**What is added by this report?**

After a decade of increasing rates, contact sports–related TBI ED visits significantly declined from 2012 to 2018. This reduction resulted primarily from a 39% decline in football-related SRR-TBIs during 2013–2018.

**What are the implications for public health practice?**

Expanding efforts to address SRR-TBIs in football, the sport with the highest incidence of TBI, and identifying prevention strategies for other sports with high rates of SRR-TBI could reduce the prevalence of these injuries among children.
